# Supplementary figures and images for: Evaluation of Sibel’s Advanced Neonatal Epidermal (ANNE) wireless continuous physiological monitor in Nairobi, Kenya
Source: PLoS One. 2022 Jun 30;17(6):e0267026. doi: 10.1371/journal.pone.0267026 (PMC9246120; doi:10.1371/journal.pone.0267026)

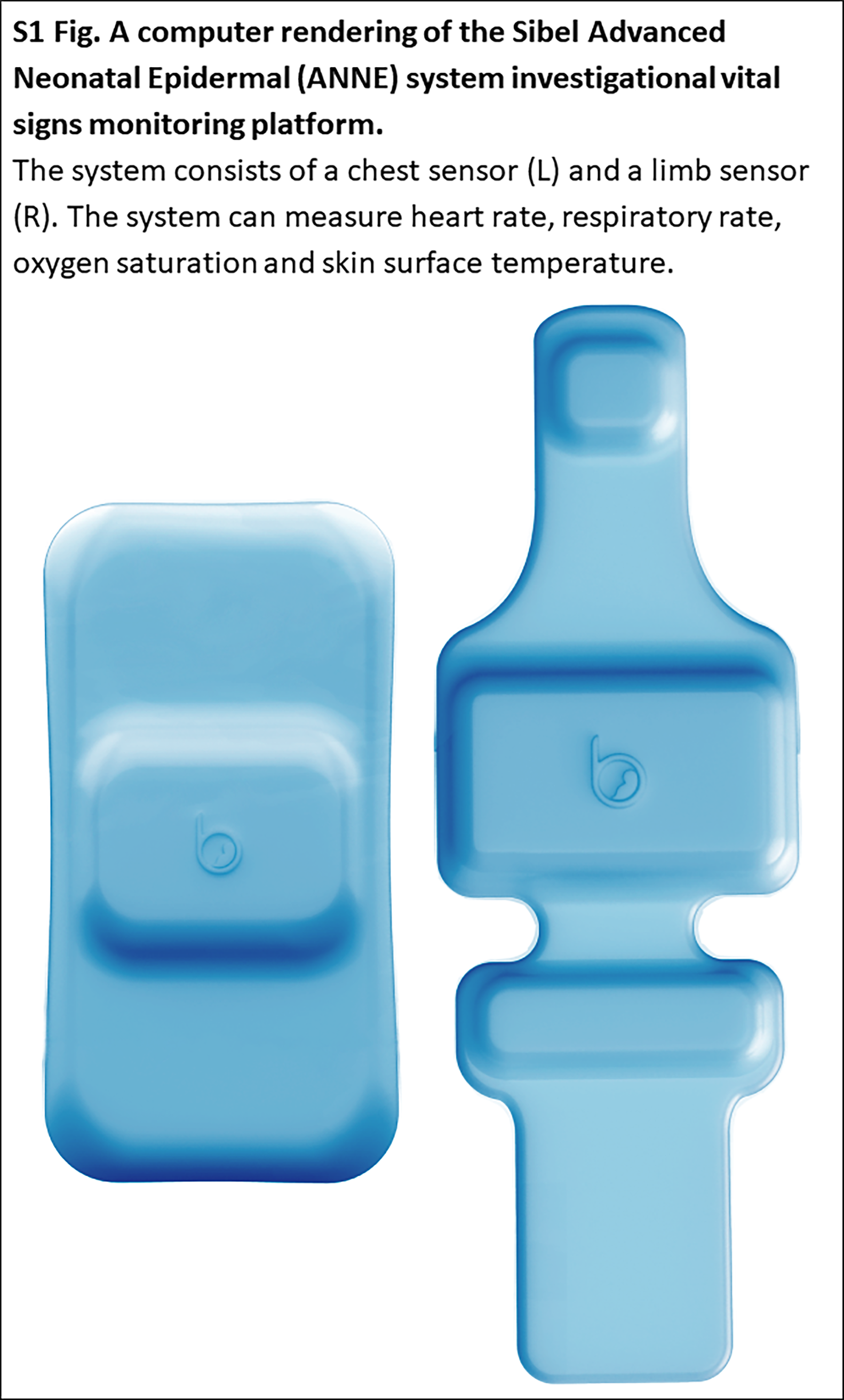

Supplement: S1 Fig — The system consists of a chest sensor (L) and a limb sensor (R). The system can respiratory rate, oxygen saturation and skin surface temperature. (TIF) [file pone.0267026.s001.tif]

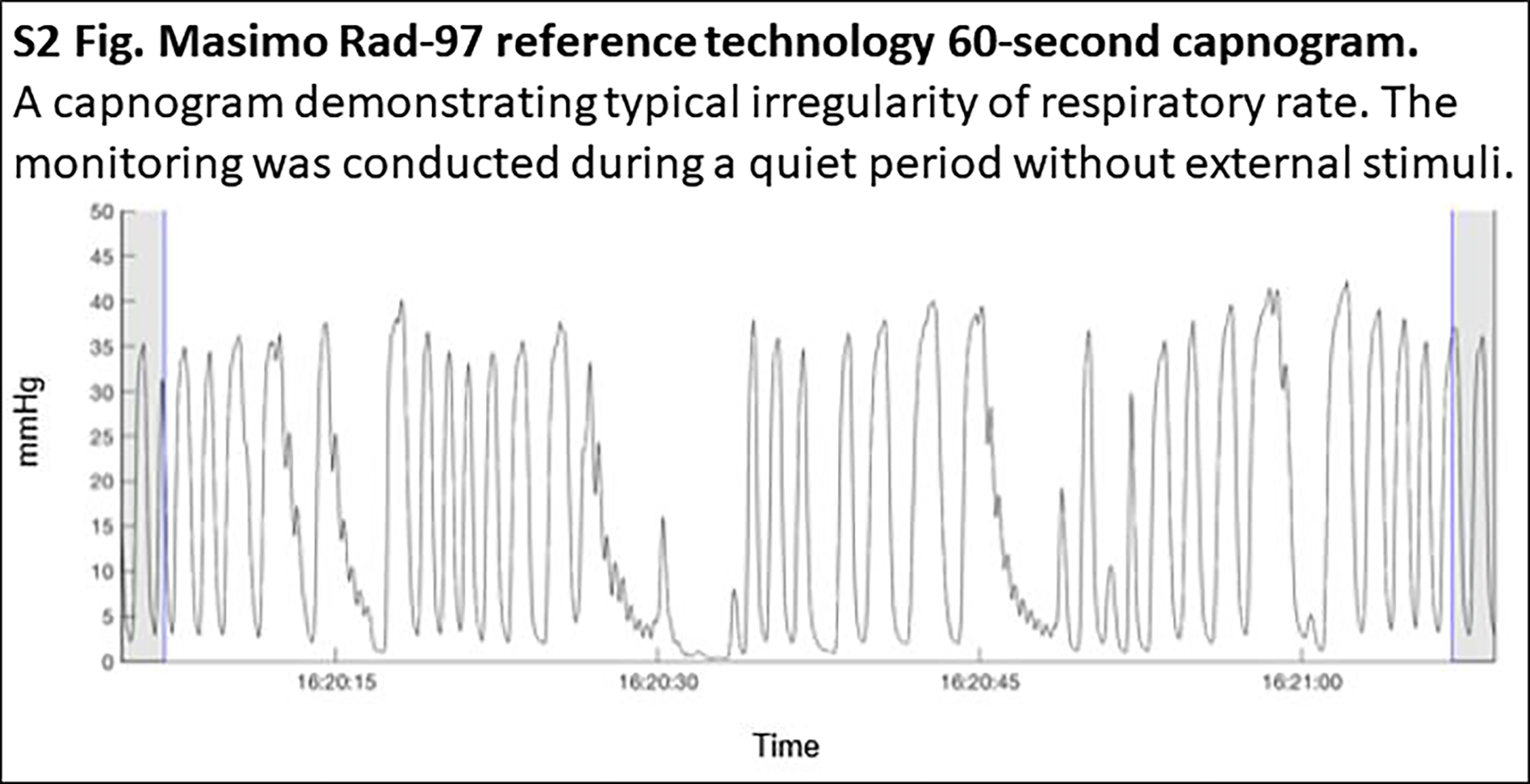

Supplement: S2 Fig — The monitoring was conducted during a quiet period without external stimuli. (TIF) [file pone.0267026.s002.tif]

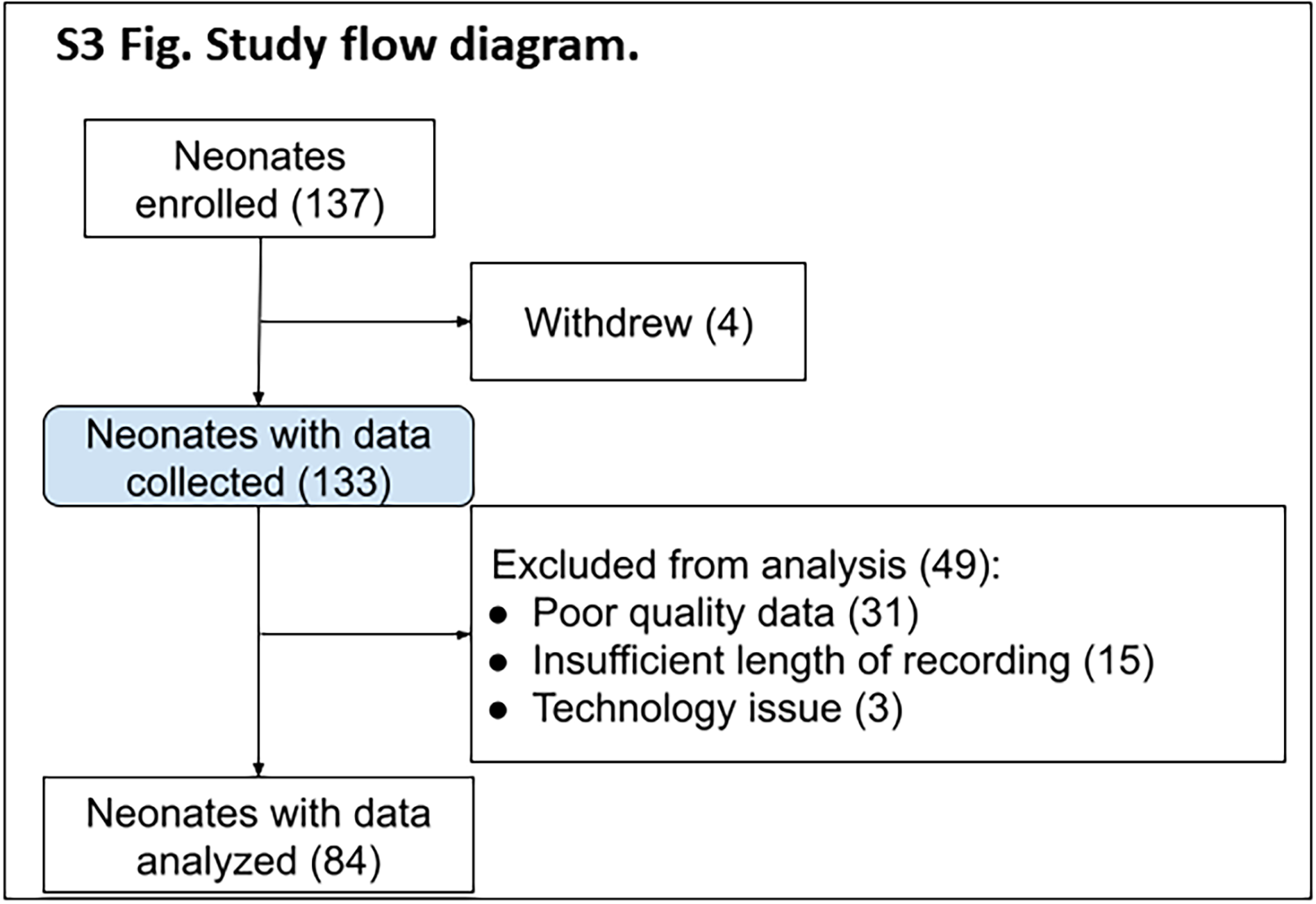

Supplement: S3 Fig — (TIF) [file pone.0267026.s003.tif]

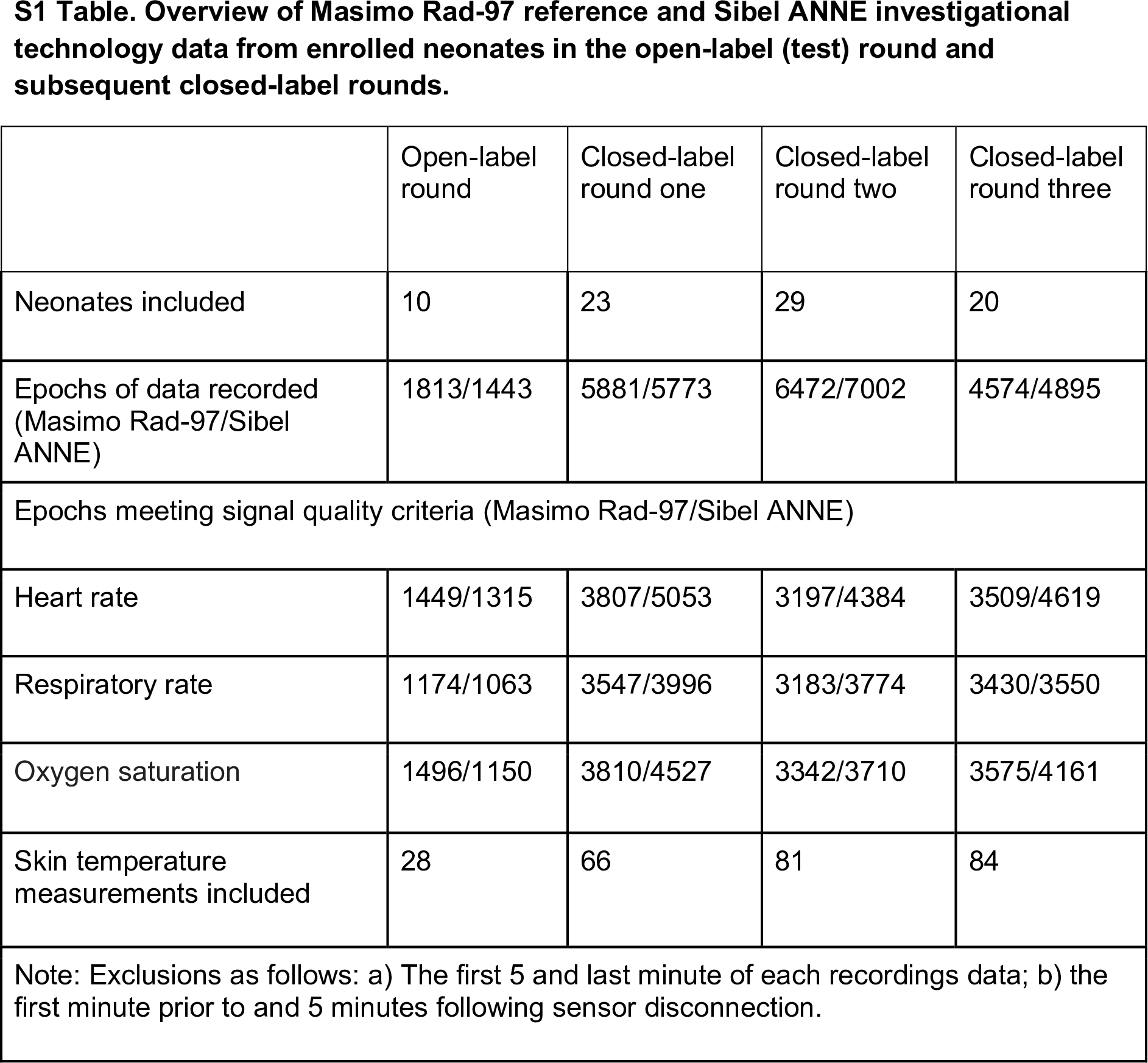

Supplement: S1 Table — (TIF) [file pone.0267026.s004.tif]
